# Factors associated with long term work incapacity following a non-catastrophic road traffic injury: analysis of a two-year prospective cohort study

**DOI:** 10.1186/s12889-022-13884-5

**Published:** 2022-08-05

**Authors:** Christopher Papic, Annette Kifley, Ashley Craig, Genevieve Grant, Alex Collie, Ilaria Pozzato, Belinda Gabbe, Sarah Derrett, Trudy Rebbeck, Jagnoor Jagnoor, Ian D. Cameron

**Affiliations:** 1 Northern Clinical School, Faculty of Medicine and Health, John Walsh Centre for Rehabilitation Research, Kolling Institute of Medican Research, The University of Sydney, Royal North Shore Hospital, Level 12, Corner Reserve Road and Westbourne Street, NSW 2065 St Leonards, Australia; 2 Australian Centre for Justice Innovation, Faculty of Law, Monash University, Clayton, Victoria 3800 Australia; 3 Healthy Working Lives Research Group, School of Public Health and Preventive Medicine, Monash University, 553 St Kilda Road, Victoria, 3004 Australia; 4 School of Public Health and Preventive Medicine, Monash University, 553 St Kilda Road, Victoria, 3004 Australia; 5 Department of Preventive and Social Medicine, University of Otago, 18 Frederick Street, Dunedin North, Dunedin, 9016 New Zealand; 6 The George Institute for Global Health, and Faculty of Medicine, University of New South Wales, Level 5/1 King St, Newtown, NSW 2042 Australia

**Keywords:** Personal injury, Motor vehicle crash, Work disability

## Abstract

**Background:**

Road traffic injuries (RTIs), primarily musculoskeletal in nature, are the leading cause of unintentional injury worldwide, incurring significant individual and societal burden. Investigation of a large representative cohort is needed to validate early identifiable predictors of long-term work incapacity post-RTI. Therefore, up until two years post-RTI we aimed to: evaluate absolute occurrence of return-to-work (RTW) and occurrence by injury compensation claimant status; evaluate early factors (e.g., biopsychosocial and injury-related) that influence RTW longitudinally; and identify factors potentially modifiable with intervention (e.g., psychological distress and pain).

**Methods:**

Prospective cohort study of 2019 adult participants, recruited within 28 days of a non-catastrophic RTI, predominantly of mild-to-moderate severity, in New South Wales, Australia. Biopsychosocial, injury, and compensation data were collected via telephone interview within one-month of injury (baseline). Work status was self-reported at baseline, 6-, 12-, and 24-months. Analyses were restricted to participants who reported paid work pre-injury (*N* = 1533). Type-3 global *p*-values were used to evaluate explanatory factors for returning to ‘any’ or ‘full duties’ paid work across factor subcategories. Modified Poisson regression modelling was used to evaluate factors associated with RTW with adjustment for potential covariates.

**Results:**

Only ~ 30% of people with RTI returned to full work duties within one-month post-injury, but the majority (76.7%) resumed full duties by 6-months. A significant portion of participants were working with modified duties (~ 10%) or not working at all (~ 10%) at 6-, 12-, and 24-months. Female sex, low education, low income, physically demanding occupations, pre-injury comorbidities, and high injury severity were negatively associated with RTW. Claiming injury compensation in the fault-based scheme operating at the time, and early identified post-injury pain and psychological distress, were key factors negatively associated with RTW up until two years post-injury.

**Conclusions:**

Long-term work incapacity was observed in 20% of people following RTI. Our findings have implications that suggest review of the design of injury compensation schemes and processes, early identification of those at risk of delayed RTW using validated pain and psychological health assessment tools, and improved interventions to address risks, may facilitate sustainable RTW.

**Trial registration:**

This study was registered prospectively with the Australian New Zealand Clinical Trials Registry (ACTRN12613000889752).

## Background

Non-catastrophic road traffic injuries (RTIs), such as musculoskeletal injury or mild traumatic brain injury (mTBI), are the leading cause of unintentional injury [1] and the sixth highest cause of disability-adjusted life years worldwide in 2019 [2]. The prevalence of hospitalization due to RTIs in Australia increased by 12.9% over the five-year period to 2018, totalling 39,598 [3] and without consideration of people who had sustained a RTI and were not hospitalized. Road traffic injuries can have detrimental long term effects on those injured, which include but are not limited to, psychological distress [4, 5], chronic pain [6], disability [7], and reduced health-related quality of life [8, 9]. In addition to individual effects, RTIs have considerable societal impact, with total societal economic burden (e.g., healthcare and loss of productivity costs) estimated at AUD29.7 billion in Australia in 2015 [10].

The physical and psychological effects of RTIs can impact a person’s work capacity, financial stability, and social productivity [7]. While it is understandable that severe orthopaedic injury, such as major lower extremity trauma, can affect a person’s ability to work [11], mild-to-moderate severity RTIs can also have long-term negative effects on work capacity. For instance, 88% of people who sustained a mTBI in a road crash had not returned to their pre-injury work capacity 6–9 months post-injury [12]. Furthermore, a pilot cohort of people who had sustained mild-to-moderate severity RTIs in NSW, found approximately one in five had not returned to paid work two years post-injury [13]. Delayed return to work (RTW) can exacerbate poor health with increased financial and psychosocial stress [14], and is associated with increased all-cause mortality [15], highlighting the need for appropriate and sustainable RTW post-RTI.

Return to work is an important indicator of recovery and real-world functioning post-injury, and engagement in work can contribute to overall health [16]. Return to work following whiplash injury, for example, was associated with greater maintenance of rehabilitation treatment gains compared with those who had not returned to work [17]. Timely RTW also promotes psychological health by enhancing social connectedness, social identity, and self-esteem [18, 19]. Determining early identifiable factors associated with work incapacity following RTI is pertinent to identifying those at risk of delayed RTW, a prerequisite to developing interventions to reduce overall injury burden [20].

Factors negatively associated with RTW following RTIs, from several Australian prospective studies, include: sociodemographic factors (e.g., older age, female sex, lower occupational skill level, lesser pre-injury paid work hours, more physically demanding occupations), pre-injury health (e.g., chronic illness), psychological factors (e.g., post-traumatic stress, depression), injury severity, and high initial pain and disability [13, 21, 22]. Additionally, involvement in injury compensation claims processes is associated with poorer post-injury physical and psychological health [23]. Poorer outcomes in compensation claimants compared with non-claimants are found to be partly mediated by injury-related disability status, psycho-physiological factors such as vulnerability to stress [24, 25], and perceived injustice [6, 26]. Evaluation of a large diverse cohort is needed to validate early identifiable factors of returning to paid work following RTI and clarify the influence of claiming injury compensation on RTW. Greater understanding of these factors may inform changes to RTW and compensation law, policy and practice, encourage early assessment strategies for people injured in road crashes, and help identify potentially modifiable factors for intervention.

The aim of this study was to evaluate factors associated with RTW following RTIs in a prospective inception cohort. To address this aim three study objectives were defined: i) to describe absolute RTW occurrence and RTW occurrence by compensation claimant status at fixed times up to two years post-RTI; ii) to establish whether early identified biopsychosocial, injury, and compensation factors are associated with RTW; and iii) to identify potentially modifiable factors (e.g., psychological distress and pain) that could be intervention targets for programs aiming to facilitate RTW after RTI.

## Methods

### Study design and recruitment procedures

A prospective inception cohort study was conducted in NSW, Australia, to evaluate Factors Influencing Social and Health outcomes of people who sustained a mild-to-moderate RTI; titled the FISH study [27]. Study details have been provided previously [27]. In summary, eligible participants were primarily identified in emergency departments from 12 hospitals, including central Sydney metropolitan (Royal North Shore Hospital and Royal Prince Alfred Hospital) and regional hospitals (Orange, Dubbo, and Bathurst health services). Additional recruitment sources (5.2% of total recruitment) were general practitioner clinics, physiotherapy clinics, and the following databases: Claims Advisory Database, and Personal Injury Registry (NSW Motor Accidents Authority, now the State Insurance Regulatory Authority).

Participant eligibility criteria were: i) ≥ 17 years old ii); within 28 days of a RTI; iii) NSW resident, or iv) sufficient English proficiency to take part in the study. Participants were excluded if they: i) had sustained major or catastrophic injuries (e.g., spinal cord injury, moderate/severe traumatic brain injury, extensive burns, major amputation); ii) had only sustained very minor soft tissue injuries (e.g., bruise, abrasion); iii) sustained an injury due to intentional self-harm; iv) death of a family member in the road traffic crash; or v) had cognitive deficits that impacted their ability to provide informed consent and participate in the study.

Eligible participants were invited to take part in the study by letter. Informed consent to participate was obtained verbally via phone for those who did not opt out. Participation involved a series of structured phone interviews; within 1-month post-injury (baseline), and follow-up interviews at 6-, 12-, and 24-months. Participants were recruited between August 2013 and December 2016; 6717 potential participants were screened, 946 refused, 3752 were beyond the to be contacted date or not reachable. 2019 people participated in the baseline interview. In the baseline interview, data were collected on participant sociodemographic characteristics, pre-injury health, injury characteristics, work status, and post-injury psychological and physical health status. These data were electronically stored on the Research Electronic Data Capture (REDCap) and Computer Assisted Diagnostic Interview platforms. Self-reported RTW status was evaluated at follow-up interviews for those who were in paid work at the time of their injury.

### Sociodemographic and pre-injury health factors

Sociodemographic and pre-injury health data were self-reported by participants during the baseline interview. Data were collected on age, sex, highest level of education, primary language spoken at home, marital status, occupation category, gross yearly income (AUD, $), and satisfaction with social relationships (5-point Likert scale: 1-poor to 5-excellent). Social satisfaction was categorized into dissatisfied (1-2), neither (3), or satisfied (4-5). The Index of Relative Socio-economic Advantage and Disadvantage (IRSAD) and Australian Bureau of Statistics assigned deciles from the 2016 Australian Census of Population and Housing were matched to postcodes where participants’ resided [28]. Pre-injury health-related quality of life was evaluated using the EQ-5D-3L measure [29]. The EQ-5D-3L assesses participants’ mobility, self-care, usual activities, pain/discomfort, and anxiety/depression with three problem severity levels (e.g., no problems, some problems, or severe problems). An overall summary index out of one was derived using Australian time trade-off derived preference weights categorised into < 0.8, 0.8- < 1.0, and full score (1.0) [30]. Pre-injury health was also evaluated according to the number of pre-existing comorbidities from those listed within the Functional Comorbidity Index [31] and body mass index (BMI, kg/m^2^) derived from self-reported body mass and height.

### Injury-related factors

Participants reported which body regions were injured, whether they presented to hospital following their RTI, and for those admitted, the length of hospitalization (days). Hospital length of stay was used as a proxy indicator of injury severity, where greater length of stay was indicative of greater injury severity; less than one day (including those not admitted to hospital), two to six days, or seven or more days, based on cut-offs determined by the International Traffic Safety Data and Analysis Group [32]. Injury severity was also evaluated using the Injury Severity Scale (ISS), derived from Abbreviated Injury Scale scores of affected body regions [33]. Injury Severity Scale scores were derived by a trained coder using methods and injury data sources described by Hung et al. [34]. Participant-perceived danger of death during the crash was evaluated on a 5-point Likert scale (0-none to 5-overwhelming). Data on whether participants had claimed injury compensation (claimant status) was obtained from the State Insurance Regulatory Authority Personal Injury Register. The NSW compulsory third party (CTP) injury compensation scheme in operation at the time was a predominantly fault-based scheme allowing people injured and not at fault in a road crash to submit a claim within six months of injury [35].

### Post-injury psychological and physical health status

Psychological and physical health status was assessed at baseline using validated questionnaires. Post-traumatic stress was evaluated using the 22-item self-reported Impact of Events Scale Revised (IES-R). The IES-R is a valid tool for measuring post-traumatic stress following a RTI [36], comprising a maximum score of four for each post-traumatic stress symptom subscales: avoidance, intrusion, and hyperarousal, which are summed to a total score out of 12. Participants with a total mean score ≥ 4.5/12 categorised as having elevated levels of post-traumatic stress, and probable post-traumatic stress disorder [4]. The 21-item Depression and Anxiety Stress Scales (DASS-21) was used to evaluate depressive mood, anxiety, and perceptions of stress [37]. A DASS-42 total score ≥ 30 out of 126 was found to be an appropriate cut-off to screen for a probable major depressive disorder following a RTI [38]. As a result, a DASS-21 cut-off of ≥ 15 out of 63 was used in our study.

Pain severity was evaluated using the numeric rating scale (NRS), on a zero to 10 scale, where zero indicated no pain and 10 represented ‘the most pain ever’ [39]. The tolerable pain threshold informed NRS pain severity classifications used in our study: no pain (zero), mild pain (1-3), and moderate to severe pain (4-10). The Örebro Musculoskeletal Pain Screening Questionnaire Short Form (OMPSQ-SF) is a 10-item psychosocial screening tool that was used to assess constructs such as anxiety, depression, fear avoidance, recovery expectations, pain and disability [40]. The OMPSQ-SF is a validated prognostic risk assessment tool where scores ≥ 50/100 stratify those at high risk of not returning to work [41] and poor health outcomes [42]. Pain related catastrophizing, including ruminating on pain, magnification of pain, and feelings of helplessness surrounding pain, was evaluated using the 13-item 5-point Likert scale tool, the Pain Catastrophizing Scale (PCS) [43]. A PCS cut off of ≥ 30/52 was used in our study to classify participants with high pain-related catastrophic thinking [43].

### Return to work status

The primary outcome was self-reported post-injury work status. Self-reported work status has been shown to be a reliable method of evaluating RTW following injury when compared with injury compensation records [44]. In our study, post-injury paid work status was classified as ‘full work duties’, ‘modified work duties’, or ‘not in paid work’, with respect to the participant’s paid work status at the time of their injury and irrespective of employment status (i.e., casual, part-time, or full-time, including self-employment). Casual employment referred to paid work on an as-needed basis without fixed hours, whilst part- and full-time employment was based on less than 38-h/week or ≥ 38-h per week, respectively [45]. Modified work duties comprised a reduction in work hours and/or modification to work tasks (e.g., lifting restrictions). As an example of RTW status classification, a participant was classified as returning to full work duties if they had returned to their pre-injury employment tasks even if they were casually employed. To evaluate factors associated with RTW at each timepoint, RTW was then re-categorized into ‘any’ (modified or full) or ‘full work duties’ RTW. It was pertinent in our study to evaluate not only factors associated with returning to full work duties, but also factors associated with any RTW as it is an important indicator of post-injury functioning and recovery.

### Statistical analyses

Analyses were restricted to participants who were in some form of paid work when their injury occurred. Pre-injury and baseline characteristics, absolute RTW occurrence, and RTW occurrence by claimant status over time were summarized using descriptive statistics. Differences in RTW status of claimants and non-claimants of compensation at each timepoint were evaluated using the Chi-square test statistic. Type 3 global test *p*-values were used to evaluate significant explanatory factors for ‘any’ and ‘full duties’ RTW across any of the subcategories of each explanatory factor. Type 3 tests of interaction terms between time point and each explanatory factor for return to full work duties were used to evaluate whether associations changed over time during the study. Modified Poisson regression modelling, with generalized estimating equations for longitudinal data from baseline to 24-months, was used to evaluate factors influencing RTW following a RTI before and after adjustment for potential covariates. This modelling accounts for missing follow-up outcome data under a missing at random assumption.

The effect of each explanatory factor at each time point from baseline to 24-months was derived from the longitudinal modelling and presented in terms of relative risk (RR) estimates with 95% confidence intervals (CI). Multivariable adjustments for preinjury factors, injury factors, baseline post-injury factors and compensation claimant status involved adjusting for other antecedent or coincident covariables. Pre-injury covariables were sex, age group, educational level, language, marital status, IRSAD (including deciles), social satisfaction, recruitment source, comorbid conditions, and pre-injury EQ-5D-3L index score. Crash/injury related covariables were crash type, hospital length of stay and perceived danger of death in the crash. Baseline post-injury covariables were IES-R total score, DASS-21 total score, pain NRS, and PCS score. Statistical significance was taken as *p* < 0.05 for all tests. Statistical analyses were performed using SAS Version 9.4 software (SAS Institute: Cary, USA).

## Results

**Fig. 1:**
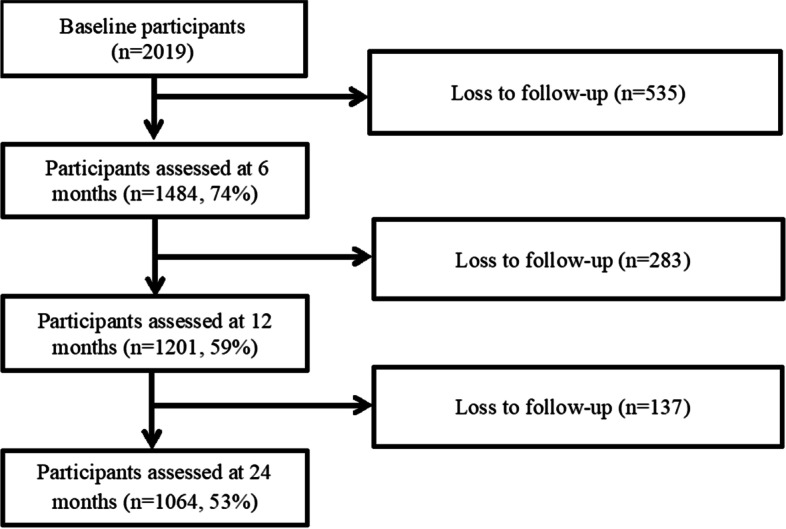
Flow chart of participant follow up rates of the entire cohort (*N* = 2019)

Figure 1 summarizes participation and follow up rates in the FISH study as a whole. Of the total cohort of 2019 participants, 1039 male and 494 female participants (mean age 39.3 ± 13.2 years, and 76% of all participants) were in paid work at the time of injury. Data from these 1533 participants were included in analyses with 72.5, 59.8, and 53.0% at 6-, 12-, and 24-months, respectively. Participant sociodemographic and pre-injury health characteristics are summarized in Table 1. Table 2 summarizes injury characteristics, and baseline (one-month post-injury) psychological and physical health status. Upper extremity, torso, and lower extremity were the most common body regions injured in road traffic crashes, with approximately one in five participants reporting injuries to each of these regions.

**Table 1:** Sociodemographic and health characteristics of study participants who were in paid work pre-injury

Characteristics	Participants (*N* = 1533)
Sociodemographic	
Post-secondary education, n (%)^a^	1062 (69.3)
English speaking, n (%)	1395 (91.0)
Marital status, n (%)	
Married/de facto	824 (53.8)
Divorced/widowed/separated	115 (7.5)
Never married	594 (38.7)
Occupation, n (%)^a^	
Professional	528 (34.4)
Clerical/administrative services	127 (8.3)
Technical/trades services	254 (16.6)
Manager	208 (13.6)
Community/personal services	135 (8.8)
Labourer	81 (5.3)
Sales worker	96 (6.3)
Machinery operator/driver	89 (5.8)
Gross yearly income, n (%)^a^	
$0-$20,799	80 (5.2)
$20,800-$41,599	231 (15.1)
$41,600-$64,999	397 (25.9)
$65,000-$103,999	412 (26.9)
$104,000 +	333 (21.7)
Social satisfaction, n (%)^a^	
Satisfied	1392 (90.8)
Neither	100 (6.5)
Dissatisfied	40 (2.6)
IRSAD, mean (SD)	1045.32 (85.77)
Pre-injury health	
EQ-5D-3L index, mean (SD)	0.94 (0.11)
Number of comorbidities, n (%)^a^	
None	744 (48.5)
1	433 (28.2)
2–3	299 (19.5)
≥ 4	56 (3.7)
BMI (kg/m^2^), n (%)^a^	
< 18.5 underweight	31 (2.0)
18.5–24.9 normal	608 (39.7)
25–29.9 overweight	543 (35.4)
30–39.9 obese	273 (17.8)

^a^Missing data: Education (*n* = 2), occupation (*n* = 15), income (*n* = 80), social satisfaction (*n* = 1), number of comorbidities (*n* = 1), and BMI (*n* = 78). *IRSAD* Index of Relative Socio-Economic Advantage and Disadvantage, EQ-5D-3L: Health-related quality of life measure, *BMI* Body Mass Index

**Table 2:** Participant injury-related characteristics, and post-injury (baseline) psychological and health status

Characteristics	Participants (*N* = 1533)
Injury-related factors	
Accident type, n (%)	
Car (driver)	508 (33.1)
Car (passenger)	119 (7.8)
Motorbike	535 (34.9)
Bicyclist	258 (16.8)
Pedestrian	94 (6.1)
Skateboard	18 (1.2)
Admitted to hospital, n (%)^a^	752 (49.1)
Recruitment site, n (%)	
Hospital (all)	1445 (94.3)
Physio/GP/online/databases	88 (5.7)
Hospital length of stay (days), n (%)^a^	
≤ 1 or no presentation to hospital	1011 (65.9)
2–6	378 (24.7)
≥ 7	143 (9.3)
Injury Severity Score, n (%)	
1–3	795 (51.9)
4–8	575 (37.5)
9–11	102 (6.7)
12 +	61 (4.0)
Perceived danger of death, n (%)^a^	
Overwhelming	134 (8.7)
Great	243 (15.9)
Moderate	294 (19.2)
Small	310 (20.2)
None	526 (34.3)
Insurance claim, n (%)	408 (26.6)
Post-injury psychological and physical health status	
IES-R ≥ 4.5/12 (elevated post-traumatic stress), n (%)	502 (32.7)
DASS-21 ≥ 15/63 (probable major depressive disorder), n (%)	454 (29.6)
Pain severity (NRS), mean (SD)	4.2 (2.6)
OMPSQ-SF ≥ 50/100 (high risk), n (%)	386 (25.2)
PCS (high ≥ 30/52), n (%)	230 (15.0)

^a^Missing data: Hospital admission information (*n* = 1) and perceived danger of death (*n* = 26). *IES-R* Impact of Events Scale Revised, *DASS-21* Depression and Anxiety Stress Scales, *NRS* Numeric Rating Scale, *OMPSQ SF* Örebro Musculoskeletal Pain Screening Questionnaire Short Form, *PCS* Pain Catastrophizing Scale

**Fig. 2:**
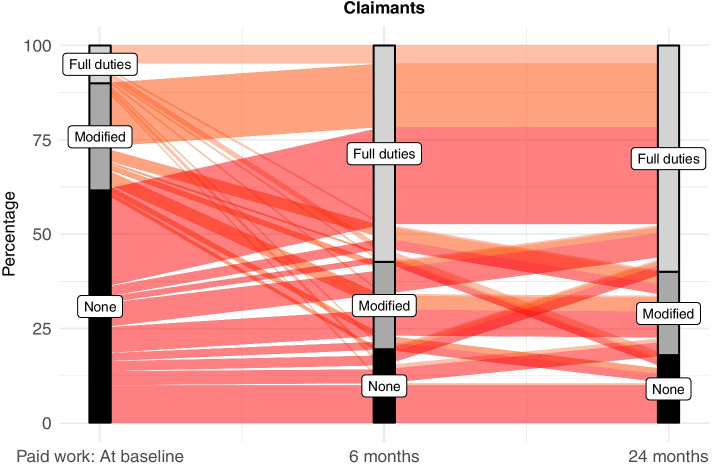
Paid work, full and modified duties, from baseline to 24-months for claimants of injury compensation

**Fig. 3:**
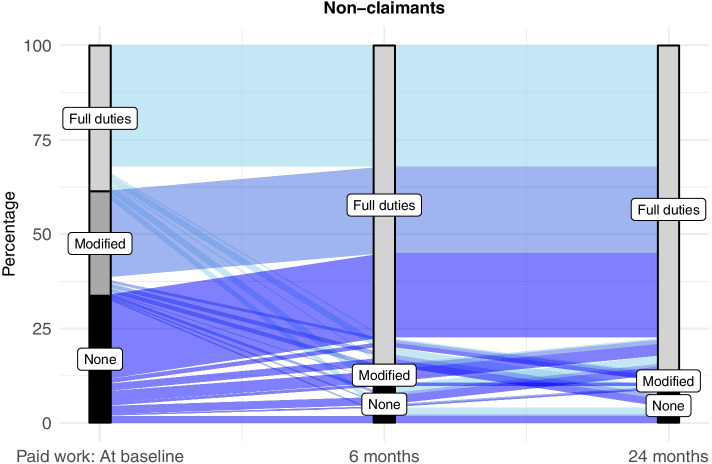
Paid work, full and modified duties, from baseline to 24-months for non-claimants of injury compensation

Table 3 displays RTW status of the cohort over time, where approximately 20% of participants had not returned to full pre-injury work duties at 6-, 12-, and 24-months post-injury, and 10% of participants were not in any form of work between 6- and 24-months post-injury. Significant differences in unadjusted RTW rates were evident between non-claimants and claimants of injury compensation at all timepoints (Table 3). The occurrence of any RTW at baseline was approximately 65% for non-claimants, compared with 40% of claimants. Disparity between these groups was evident for full duties RTW longitudinally, with ~ 85% of non-claimants working in full duties at all follow up time points compared with ~ 60% of claimants; notably 20% of claimants were not in full duties work 24-months post-injury. Figs. 2 and 3 illustrate changes in RTW status over time for claimants (*n* = 190) and non-claimants (*n* = 520) of injury compensation who had complete follow-up data at baseline, 6-, and 24-months post-injury (note: 12-month data were omitted from these figures as it did not provide further information in addition to the 24-month work status data).

**Table 3:** Occurrence of return to work of participants who were working at the time of their injury, and occurrence by compensation status subgroups, at fixed time points up to two years post-RTI

Return to work status	n (%)			
	Baseline	6-months	12-months	24-months
**All participants**	(*N* = 1533)	(*N* = 1111)	(*N* = 916)	(*N* = 812)
Full duties	457 (29.8)	852 (76.7)	727 (79.4)	635 (78.2)
Modified duties	437 (28.5)	121 (10.9)	84 (9.2)	75 (9.2)
Not working	620 (40.4)	131 (11.8)	97 (10.6)	97 (11.9)
Missing data	19 (1.2)	7 (0.6)	8 (0.1)	5 (0.6)
**Claimed compensation**	(*N* = 408)	(*N* = 298)	(*N* = 236)	(*N* = 222)
Full duties	35 (8.6)	154 (51.7)	147 (62.3)	133 (59.9)
Modified duties	125 (30.6)	77 (25.8)	57 (24.2)	48 (21.6)
Not working	245 (60.0)	62 (20.8)	31 (13.1)	41 (18.5)
Missing data	3 (0.3)	5 (1.7)	1 (0.4)	-
**Did not claim compensation**	(*N* = 1125)	(*N* = 813)	(*N* = 680)	(*N* = 590)
Full duties	422 (37.5)	698 (85.9)	580 (85.3)	502 (85.1)
Modified duties	312 (27.7)	44 (5.4)	27 (4.0)	27 (4.6)
Not working	375 (37.5)	69 (8.5)	66 (9.7)	56 (9.5)
Missing data	16 (1.4)	2 (0.2)	7 (1.0)	5 (0.8)
***Χ***^**2**^, ***p*-value**	139.03, *p* < *0.001*	145.51, *p* < 0.001	90.16, *p* < 0.001	76.66, *p* < 0.001

*Χ*^2^: Pearson Chi-square test of RTW occurrence between claimants and non-claimants of injury compensation

After multivariate adjustment, significant sociodemographic, pre-injury health, injury, and post-injury psychological and physical health explanatory factors of any and full work duties RTW following a RTI were found, and most of these associations changed with time post-injury as shown by a significant interaction term (Table 4).

**Table 4:** Presence of any multivariable-adjusted association between potential explanatory factors and return-to-work following road traffic injury

Explanatory factors	Type 3 global *p*-value		
	Any RTW	Full duties RTW	Full duties RTW: Interaction with time point*
Sociodemographic			
Sex	-	-	0.020
Education	-	0.030	0.003
Language	-	0.013	-
Occupation	-	< 0.001	< 0.001
Income	0.001	-	0.03
Pre-injury health			
Number of comorbidities	0.003	-	-
Injury factors			
Crash type	0.007	0.026	< 0.001
Admission to hospital	0.015	< 0.001	< 0.001
Hospital length of stay	0.009	< 0.001	< 0.001
ISS	0.02	< 0.001	< 0.001
Perceived danger (death)	-	-	0.015
Insurance claim	0.001	< 0.001	< 0.001
Post-injury psychological/physical health status			
Pain severity	0.001	< 0.001	< 0.001
PCS	0.008	0.019	0.002
DASS-21	< 0.001	0.007	0.002
IES-R	-	-	0.024
OMPSQ-SF	0.002	< 0.001	< 0.001

^*^Overall *p*-value for the presence of effect modification between effects of the explanatory factor and time post-injury. *PCS* Pain Catastrophizing Scale, *DASS-21* Depression and Anxiety Stress Scales, *IES-R* Impact of Events Scale Revised, *OMPSQ-SF* Örebro Musculoskeletal Pain Screening Questionnaire Short Form

Table 5 shows multivariate adjusted potential factors associated with RTW at fixed times up to two years post-injury. Significant multivariate adjusted associations were found across diverse explanatory factors and time, including at 12 and 24-months post-injury. Female sex, low education, low income, physically demanding occupations (e.g., labourers and trades services), and pre-injury comorbidities were negatively associated with RTW. Injury severity, claiming injury compensation, and post-injury psychological and physical health factors were more consistently associated with RTW up until two years post-injury, compared with sociodemographic and pre-injury health factors. Participants who sustained injuries in motorcycle and pedestrian/skateboard crashes were less likely to have returned to full work duties at baseline compared with drivers injured in car crashes. Admission to hospital reduced the likelihood of returning to full work duties at baseline by ~ 70% compared with no hospital admission. Greater injury severity, indicated by greater hospital length of stay and ISS versus one or no days in hospital and mild injury, was largely associated with reduced RTW at baseline and 6-months. Severe injuries (ISS ≥ 12), however, were found to impact capacity for returning to full work duties up to 24-months.

**Table 5:** Multivariable-adjusted associations between potential explanatory factors and return-to-work status post road traffic injury

Outcome variables	Multivariate adjusted relative risk (95%CI)						
	Baseline	6-months		12-months		24-months	
	Full duties RTW	Any RTW	Full duties RTW	Any RTW	Full duties RTW	Any RTW	Full duties RTW
Sociodemographic							
Age (years)							
45–59	1.00 (ref)†	1.00 (ref)	1.00 (ref)	1.00 (ref)	1.00 (ref)	1.00 (ref)	1.00 (ref)
17–24	1.17 (0.92–1.49)	0.89* (0.82–0.97)	0.94 (0.83–1.05)	0.96 (0.89–1.03)	1.03 (0.93–1.15)	0.94 (0.84–1.05)	1.02 (0.90–1.15)
25–44	1.14 (0.95–1.37)	0.99 (0.95–1.04)	1.01 (0.94–1.08)	0.98 (0.94–1.03)	1.04 (0.97–1.11)	1.03 (0.98–1.08)	1.05 (0.97–1.13)
60–69	1.17 (0.82–1.67)	0.93 (0.84–1.04)	0.86 (0.71–1.04)	0.94 (0.84–1.05)	0.96 (0.81–1.13)	0.92 (0.81–1.04)	0.79* (0.63–0.98)
≥ 70	0.46 (0.12–1.74)	0.82 (0.59–1.13)	0.76 (0.47–1.22)	0.85 (0.64–1.13)	0.68 (0.41–1.12)	0.92 (0.72–1.19)	0.71 (0.44–1.15)
Sex							
Female	1.00 (ref)	1.00 (ref)	1.00 (ref)	1.00 (ref)	1.00 (ref)	1.00 (ref)	1.00 (ref)
Male	0.92 (0.78–1.08)	1.05 (1.00–1.11)	1.15* (1.07–1.25)	1.05 (0.99–1.10)	1.05 (0.97–1.12)	1.07* (1.00–1.13)	1.12 (1.03–1.22)
Education							
Tertiary/university	1.00 (ref)	1.00 (ref)	1.00 (ref)	1.00 (ref)	1.00 (ref)	1.00 (ref)	1.00 (ref)
Technical/other	0.70*** (0.57–0.85)	0.97 (0.92–1.02)	0.93 (0.86–1.01)	1.01 (0.96–1.06)	0.98 (0.91–1.06)	0.98 (0.93–1.04)	1.04 (0.96–1.12)
Secondary	0.72** (0.59–0.87)	0.90** (0.85–0.96)	0.90 (0.82–0.98)	0.96 (0.90–1.01)	0.92 (0.85–1.01)	0.91* (0.84–0.98)	0.91 (0.83–1.01)
Primary/pre-primary	0.66 (0.42–1.04)	0.91 (0.79–1.06)	0.93 (0.77–1.13)	0.81 (0.64–1.03)	0.83 (0.61–1.13)	1.07 (0.93–1.24)	1.00 (0.79–1.25)
Language							
English	1.00 (ref)	1.00 (ref)	1.00 (ref)	1.00 (ref)	1.00 (ref)	1.00 (ref)	1.00 (ref)
Non English	0.71 (0.51–0.98)	0.95 (0.87–1.05)	0.76* (0.65–0.91)	0.96 (0.87–1.06)	0.80 (0.68–0.94)	0.99 (0.90–1.08)	0.79 (0.65–0.95)
Marital status							
Married/defacto	1.00 (ref)	1.00 (ref)	1.00 (ref)	1.00 (ref)	1.00 (ref)	1.00 (ref)	1.00 (ref)
Divorced/widowed/separated	0.86 (0.61–1.22)	0.97 (0.89–1.06)	0.96 (0.84–1.10)	0.97 (0.88–1.07)	1.04 (0.90–1.20)	0.98 (0.88–1.08)	0.94 (0.79–1.11)
Never married	1.06 (0.90–1.24)	0.95 (0.91–1.00)	0.95 (0.89–1.02)	0.97 (0.92–1.02)	0.97 (0.91–1.05)	0.96 (0.91–1.02)	0.99 (0.92–1.07)
Occupation							
Professional	1.00 (ref)	1.00 (ref)	1.00 (ref)	1.00 (ref)	1.00 (ref)	1.00 (ref)	1.00 (ref)
Clerical/admin services	1.03 (0.80–1.34)	0.90 (0.81–1.00)	0.93 (0.81–1.08)	1.01 (0.94–1.09)	1.00 (0.89–1.14)	1.02 (0.93–1.12)	1.04 (0.90–1.20)
Technical/trades services	0.50*** (0.38–0.66)	0.98 (0.92–1.04)	0.90 (0.81–1.00)	1.00 (0.94–1.06)	0.94 (0.85–1.04)	1.06 (0.99–1.13)	1.05 (0.96–1.16)
Manager	0.91 (0.73–1.13)	1.04 (0.99–1.09)	1.09** (1.01–1.17)	1.02 (0.97–1.08)	1.02 (0.94–1.11)	1.02 (0.95–1.10)	1.05 (0.95–1.15)
Community/personal services	0.78 (0.58–1.05)	0.95 (0.86–1.04)	0.96 (0.85–1.10)	0.91 (0.81–1.01)	0.92 (0.80–1.06)	1.00 (0.89–1.12)	1.05 (0.91–1.20)
Labourer	0.42*** (0.24–0.71)	0.79** (0.67–0.94)	0.81 (0.66–1.00)	0.83* (0.71–0.98)	0.88 (0.72–1.08)	1.03 (0.88–1.20)	1.16 (0.98–1.37)
Sales worker	0.68 (0.47–0.99)	0.91 (0.80–1.02)	0.91 (0.77–1.07)	0.97 (0.87–1.08)	0.97 (0.83–1.12)	0.94 (0.82–1.08)	0.97 (0.82–1.15)
Machinery operator/driver	0.69 (0.47–1.02)	0.92 (0.82–1.03)	1.00 (0.85–1.17)	0.92 (0.81–1.04)	0.99 (0.83–1.17)	1.02 (0.87–1.19)	0.85 (0.66–1.10)
Gross yearly income (AUD)							
≥ $104,000	1.00 (ref)	1.00 (ref)	1.00 (ref)	1.00 (ref)	1.00 (ref)	1.00 (ref)	1.00 (ref)
$65,000-$103,999	1.02 (0.83–1.25)	0.98 (0.93–1.03)	0.97 (0.91–1.05)	1.00 (0.95–1.05)	0.95 (0.88–1.02)	1.02 (0.97–1.08)	1.06 (0.98–1.15)
$41,600-$64,999	0.87 (0.69–1.08)	0.94 (0.89–0.99)	0.90 (0.82–0.99)	0.96 (0.91–1.02)	0.96 (0.88–1.05)	0.97 (0.92–1.04)	1.06 (0.96–1.16)
$20,800-$41,599	0.80 (0.60–1.05)	0.97 (0.91–1.04)	0.92 (0.81–1.03)	1.02 (0.95–1.08)	0.96 (0.86–1.07)	0.96 (0.87–1.07)	0.95 (0.82–1.10)
$0-$20,799	1.13 (0.80–1.61)	0.68*** (0.56–0.83)	0.72 (0.57–0.90)	0.77** (0.65–0.92)	0.70 (0.55–0.89)	0.81* (0.68–0.97)	0.84 (0.67–1.06)
Social satisfaction							
Satisfied	1.00 (ref)	1.00 (ref)	1.00 (ref)	1.00 (ref)	1.00 (ref)	1.00 (ref)	1.00 (ref)
Neither	0.75 (0.52–1.10)	0.99 (0.89–1.09)	1.11 (0.98–1.25)	0.95 (0.86–1.06)	0.88 (0.75–1.04)	0.98 (0.86–1.12)	1.01 (0.87–1.18)
Dissatisfied	0.88 (0.52–1.50)	1.01 (0.88–1.17)	0.94 (0.76–1.17)	0.96 (0.82–1.12)	0.96 (0.78–1.18)	0.98 (0.82–1.16)	1.00 (0.78–1.27)
Pre-injury health							
EQ-5D-3L							
> 0.9	1.00 (ref)	1.00 (ref)	1.00 (ref)	1.00 (ref)	1.00 (ref)	1.00 (ref)	1.00 (ref)
> 0.8–0.9	1.33 (1.01–1.75)	0.99 (0.91–1.08)	1.04 (0.92–1.17)	0.95 (0.87–1.05)	0.96 (0.84–1.11)	0.94 (0.83–1.06)	0.91 (0.78–1.07)
≤ 0.8	1.08 (0.89–1.31)	0.94 (0.88–1.00)	0.94 (0.86–1.04)	0.91** (0.85–0.97)	0.94 (0.85–1.03)	0.97 (0.90–1.04)	1.00 (0.91–1.10)
No. comorbidities							
None	1.00 (ref)	1.00 (ref)	1.00 (ref)	1.00 (ref)	1.00 (ref)	1.00 (ref)	1.00 (ref)
1	1.02 (0.85–1.21)	0.96 (0.91–1.01)	0.96 (0.90–1.03)	0.97 (0.92–1.02)	0.94 (0.88–1.01)	0.97 (0.92–1.03)	0.94 (0.87–1.01)
2–3	0.88 (0.71–1.10)	0.91** (0.85–0.98)	0.86 (0.78–0.96)	0.91** (0.85–0.97)	0.88 (0.80–0.97)	0.91* (0.84–0.98)	0.85 (0.76–0.94)
≥ 4	1.00 (0.66–1.52)	1.01 (0.91–1.12)	0.96 (0.79–1.16)	0.98 (0.89–1.10)	0.83 (0.66–1.04)	0.95 (0.83–1.08)	0.84 (0.67–1.03)
BMI (kg/m^2^)							
< 30	1.00 (ref)	1.00 (ref)	1.00 (ref)	1.00 (ref)	1.00 (ref)	1.00 (ref)	1.00 (ref)
≥ 30 (obese)	0.96 (0.78–1.18)	0.99 (0.93–1.05)	1.01 (0.93–1.10)	0.99 (0.94–1.05)	0.97 (0.88–1.06)	1.00 (0.93–1.06)	0.99 (0.90–1.09)
Injury-related factors							
Crash type							
Car (driver)	1.00 (ref)†	1.00 (ref)	1.00 (ref)	1.00 (ref)	1.00 (ref)	1.00 (ref)	1.00 (ref)
Car (passenger)	0.87 (0.65–1.16)	0.89 (0.78–1.01)	0.83 (0.70–0.99)	0.98 (0.88–1.10)	0.97 (0.82–1.14)	0.95 (0.83–1.09)	0.95 (0.80–1.12)
Motorbike	0.65*** (0.54–0.79)	1.03 (0.97–1.08)	1.06 (0.98–1.16)	1.02 (0.97–1.08)	1.10** (1.01–1.20)	1.02 (0.96–1.09)	1.09 (0.99–1.20)
Bicyclist	0.99 (0.82–1.19)	1.05 (0.99–1.11)	1.04 (0.96–1.13)	1.06* (1.00–1.12)	1.02 (0.93–1.12)	1.09** (1.02–1.15)	1.06 (0.97–1.16)
Pedestrian/skateboard	0.68* (0.49–0.95)	0.94 (0.84–1.05)	0.82 (0.69–0.97)	0.97 (0.86–1.08)	0.88 (0.74–1.05)	0.91 (0.78–1.05)	0.83 (0.68–1.02)
Hospital admission							
No	1.00 (ref)	1.00 (ref)	1.00 (ref)	1.00 (ref)	1.00 (ref)	1.00 (ref)	1.00 (ref)
Yes	0.31*** (0.26–0.38)	0.92*** (0.88–0.96)	0.87 (0.82–0.93)	0.95 (0.91–0.99)	0.93 (0.88–1.00)	0.98 (0.93–1.03)	0.96 (0.90–1.03)
Recruitment source site							
(Hospital) Sydney metropolitan	1.00 (ref)	1.00 (ref)	1.00 (ref)	1.00 (ref)	1.00 (ref)	1.00 (ref)	1.00 (ref)
(Hospital) Regional	0.46*** (0.33–0.64)	1.04 (0.98–1.11)	1.10 (0.98–1.22)	1.03 (0.97–1.10)	1.09 (0.97–1.22)	1.06 (1.00–1.14)	1.19* (1.06–1.34)
(Hospital) Other	0.81* (0.66–0.98)	0.97 (0.91–1.03)	0.97 (0.88–1.07)	1.00 (0.94–1.06)	1.02 (0.93–1.12)	0.96 (0.89–1.04)	1.04 (0.94–1.15)
Physio/GP/online/databases	0.76 (0.53–1.10)	1.07 (0.99–1.14)	0.94 (0.79–1.11)	1.07 (1.00–1.15)	1.05 (0.90–1.22)	1.02 (0.92–1.14)	0.96 (0.80–1.15)
Hospital length of stay (days)							
≤ 1 or no presentation to hospital	1.00 (ref)	1.00 (ref)	1.00 (ref)	1.00 (ref)	1.00 (ref)	1.00 (ref)	1.00 (ref)
2–6	0.33*** (0.25–0.44)	0.98 (0.93–1.03)	0.93 (0.86–1.00)	0.97 (0.92–1.02)	0.95 (0.88–1.02)	0.97 (0.91–1.03)	0.93 (0.86–1.01)
≥ 7	0.02*** (0.00–0.14)	0.77*** (0.67–0.88)	0.65*** (0.53–0.79)	0.89 (0.80–0.99)	0.77 (0.64–0.93)	0.94 (0.84–1.06)	0.90 (0.76–1.05)
Injury Severity Score							
1–3	1.00 (ref)	1.00 (ref)	1.00 (ref)	1.00 (ref)	1.00 (ref)	1.00 (ref)	1.00 (ref)
4–8	0.40*** (0.33–0.48)	0.98 (0.94–1.02)	0.93* (0.88–1.00)	0.96 (0.92–1.01)	0.93* (0.87–1.00)	0.97 (0.92–1.02)	0.99 (0.92–1.07)
9–11	0.19*** (0.10–0.36)	0.93 (0.84–1.03)	0.83* (0.71–0.98)	0.98 (0.90–1.06)	0.92 (0.80–1.07)	0.95 (0.86–1.05)	0.94 (0.80–1.10)
≥ 12	< 0.001***	0.74** (0.60–0.92)	0.62** (0.46–0.83)	0.83 (0.69–1.00)	0.65** (0.49–0.86)	0.86 (0.72–1.03)	0.77* (0.60–0.99)
Perceived danger of death							
None	1.00 (ref)	1.00 (ref)	1.00 (ref)	1.00 (ref)	1.00 (ref)	1.00 (ref)	1.00 (ref)
Small	1.03 (0.86–1.23)	1.00 (0.95–1.05)	0.98 (0.91–1.06)	1.03 (0.98–1.08)	1.05 (0.98–1.13)	1.05 (0.99–1.11)	1.06 (0.98–1.15)
Moderate	0.74 (0.59–0.93)	0.97 (0.92–1.03)	0.99 (0.90–1.07)	0.98 (0.93–1.04)	1.07 (0.98–1.16)	0.99 (0.92–1.06)	1.06 (0.96–1.17)
Great	0.67* (0.52–0.87)	0.89** (0.82–0.96)	0.93 (0.84–1.03)	0.89* (0.81–0.97)	0.90 (0.80–1.02)	0.98 (0.90–1.07)	0.98 (0.87–1.11)
Overwhelming	0.70 (0.50–0.97)	0.93 (0.85–1.03)	0.85 (0.73–0.99)	0.99 (0.91–1.07)	0.93 (0.79–1.09)	0.95 (0.85–1.05)	0.92 (0.79–1.08)
Insurance claim							
No	1.00 (ref)	1.00 (ref)	1.00 (ref)	1.00 (ref)	1.00 (ref)	1.00 (ref)	1.00 (ref)
Yes	0.27*** (0.19–0.38)	0.90*** (0.84–0.95)	0.76*** (0.68–0.84)	0.96 (0.91–1.02)	0.87** (0.79–0.96)	0.91** (0.85–0.97)	0.84** (0.76–0.93)
Post-injury psychological and physical health status							
IES-R							
Normal (< 4.5)	1.00 (ref)	1.00 (ref)	1.00 (ref)	1.00 (ref)	1.00 (ref)	1.00 (ref)	1.00 (ref)
Elevated post-traumatic stress (≥ 4.5)	0.83 (0.68–1.01)	0.95 (0.90–1.00)	1.11* (1.01–1.22)	0.96 (0.91–1.02)	1.10 (1.00–1.22)	0.96 (0.90–1.03)	1.11 (1.00–1.24)
DASS-21							
≤ 15	1.00 (ref)	1.00 (ref)	1.00 (ref)	1.00 (ref)	1.00 (ref)	1.00 (ref)	1.00 (ref)
Probable major depressive disorder (≥ 15)	0.66*** (0.53–0.81)	0.90** (0.85–0.96)	0.96 (0.86–1.06)	0.90** (0.84–0.96)	0.94 (0.84–1.05)	0.90** (0.84–0.97)	0.98 (0.88–1.10)
Pain severity (NRS)							
No pain (0)	1.00 (ref)	1.00 (ref)	1.00 (ref)	1.00 (ref)	1.00 (ref)	1.00 (ref)	1.00 (ref)
Mild (1–3)	0.67*** (0.58–0.78)	1.00 (0.94–1.06)	1.08 (1.00–1.18)	1.01 (0.96–1.06)	1.05 (0.97–1.13)	1.04 (0.97–1.11)	1.09* (1.01–1.19)
Moderate-severe (4–10)	0.37*** (0.31–0.44)	**0.95 (0.90–1.01)**	1.07 (0.98–1.17)	0.95 (0.90–1.00)	1.03 (0.95–1.11)	0.95 (0.88–1.01)	1.04 (0.95–1.14)
OMPSQ-SF							
< 50	1.00 (ref)	1.00 (ref)	1.00 (ref)	1.00 (ref)	1.00 (ref)	1.00 (ref)	1.00 (ref)
High risk (≥ 50)	0.26*** (0.17–0.40)	0.87*** (0.81–0.94)	0.81** (0.70–0.93)	0.92* (0.85–0.99)	0.82** (0.70–0.95)	0.90* (0.83–0.98)	0.92 (0.79–1.06)
PCS							
< 30	1.00 (ref)	1.00 (ref)	1.00 (ref)	1.00 (ref)	1.00 (ref)	1.00 (ref)	1.00 (ref)
High pain-related catastrophic thinking (≥ 30)	0.52*** (0.35–0.76)	0.85** (0.76–0.94)	0.96 (0.81–1.14)	0.95 (0.87–1.05)	0.99 (0.83–1.19)	0.85** (0.75–0.96)	0.98 (0.81–1.18)

^†^: Reference variable

Significant associations after additional *p*-value adjustments for all factors are indicated in bold: *p < 0.05, **p < 0.01, ***p < 0.001

*RTW* Return-to-work, *IES-R* Impact of Events Scale Revised, *DASS-21* Depression and Anxiety Stress Scales, *NRS* Numeric Rating Scale, *OMPSQ-SF* Örebro Musculoskeletal Pain Screening Questionnaire Short Form, *PCS* Pain Catastrophizing Scale

Key factors that were negatively associated with RTW over the 24-months included: making an injury compensation claim, early identified post-injury pain and early psychological distress, assessed by tools such as the OMPSQ-SF and DASS-21. Participants who claimed injury compensation were significantly less likely to RTW at all timepoints compared with non-claimants. Claiming compensation had a larger association with returning to full work duties compared with any RTW, notably reducing the likelihood of returning to full work duties by 73% at baseline and 24% at 6-months. Pain severity was a significant predictor of RTW at baseline only, with individuals with mild or moderate-severe pain 33 and 63% less likely than those with no pain to return to full work duties, respectively. Participants who exhibited probable major depressive disorder (DASS-21), pain related disability and psychological distress (OMPSQ-SF), or pain related catastrophizing thinking (PCS), versus those who did not exhibit elevated symptoms in each of these psychometric subscales, were more likely to exhibit long-term work incapacity.

## Discussion

We have shown that while only 30% of people who had sustained mild-to-moderate injuries in a road traffic crash return to full work duties within one-month post-injury, the majority (77%) resume full work duties by 6-months. However, a significant portion of participants of this cohort (~ 20%) were working with modified duties or not working at all; a consistent finding at 6-, 12-, and 24-months post-RTI. A range of explanatory biopsychosocial and injury factors were found to be associated with RTW, which may account for delayed RTW and modified work capacity within this cohort. Claiming injury compensation in a fault-based scheme was a key predictor of reduced RTW likelihood at fixed time points up to two years post-RTI. Early identified pain and psychological distress were negatively associated with RTW longitudinally. These findings have implications for early detection of those at risk of long-term work incapacity following RTI, and for targeting these factors with appropriate intervention.

### Return to work occurrence

Return to work rates following injury vary within compensable settings [46, 47]. The occurrence of any RTW was slightly higher in our cohort two years post-injury injury (~ 90%) than a previous NSW cohort under the same compensation scheme, where 82% of participants were in some form of paid work two years post-RTI [13]. In a combined cohort of participants recruited from three injury compensation schemes across NSW and Victoria, Australia, 64–75% were in paid work two years post-RTI [48]. A lower RTW rate in the combined cohort may be partly attributed to injury severity, with ~ 70% of the combined cohort having sustained severe injuries (ISS ≥ 9), compared with 52 and 38% of participants in our cohort having sustained mild or moderate injuries, respectively. Return to work occurrence rate variability is also observed when comparing RTIs to workplace injuries. 77% of participants in our cohort had returned to full work duties 6-months post-injury, compared with: 75% of workers 6-months after workplace or road injuries requiring hospitalization in Victoria, Australia [49]; 83.6% of workers 7-months after occupational injury in China [50]; and 66.6% of workers 6-months following orthopaedic injury in Taiwan [51]. Comparing RTW occurrence between these studies, however, can be problematic due to sample differences, jurisdictional differences in compensation schemes, and different methods of calculating RTW status [20].

Return to work rates in our cohort were established by 6-months and remained constant until two years post-injury. This trend was noted by Giummarra et al. in compensable injury settings (workplace and RTIs) [48], and was also observed following major traumatic injury (e.g., falls, RTI) [52]. One in five participants in our cohort were working with modified duties or not working at all two years post-injury, which not only affect the individual (e.g., health-related quality of life [53]), but can also impact the wider population. Injury-related delayed work may partly explain the high economic burden of RTIs in NSW [54], due to injury related disability and factors such as compensation, healthcare utilization costs, and economic production losses [55]. Given that RTW rates remained constant 6-months following RTI, targeting those not in work or working with modified duties with early and appropriate intervention may reduce long term individual and societal burden.

### Claiming compensation

While injury compensation schemes are developed to provide income support, and assist recovery, claiming compensation has been found to be associated with poor health outcomes and work disability [23, 47, 56]. The design of compensation schemes, including the benefits available and the way they are claimed and delivered, can impact on claimants’ health and work outcomes [57]. Return to work occurrence in our cohort was significantly lower in claimants at all timepoints compared with non-claimants and claimants were more likely to be working in modified duties at all timepoints. It is difficult to discern whether participants who could not return to work immediately after injury had a higher propensity to claim compensation, or whether compensation scheme factors contributed to delayed RTW. Claiming compensation was shown to be associated with reduced likelihood for RTW at all timepoints after accounting for covariates that include pre-injury health, disability, and psychological status, as people who have poorer outcomes under these domains are shown to be more likely to claim compensation and exhibit long-term work incapacity [58].

Claiming compensation was associated with reduced likelihood of returning to full work duties more so than any RTW, suggestive of compensation scheme design effects. Under this scheme there was no payment for wage loss until the end of the claim (except in situations of severe financial hardship) and RTW was not a priority until it was clear that it would be delayed. We note prior research reporting that administrative processes, including the requirement for medical assessments and documentation [59], and requirement for the person injured to prove the legitimacy of their claim [60] contribute to delays in RTW and recovery. There is strong evidence that claiming compensation is associated with chronic pain [61], and poor psychological outcomes [23]. These associations are mitigated by factors such as secondary victimization [62] and perceived injustice [26] which are common within fault-based schemes [63]. A systematic review of fault related legal and compensation procedures after RTIs concluded that there was limited evidence of poorer work-related outcomes in fault-based compensation schemes [56]. Our study supports the assertion that claiming compensation within a fault-based compensation scheme was negatively associated with RTW, though the mechanisms involved require further investigation: the role of stress vulnerability and injury-related disability has been shown to play an important role in any claimant distress [26], for example.

### Factors associated with return to work

Sociodemographic and injury factors associated with RTW were consistent with previous injury-related research. For example, female sex, lower levels of education, and lower income were associated with reduced likelihood of RTW over time, which is consistent across a range of injury causes and settings [13, 64, 65]. Employment requiring physical tasks, such as technical/trade services and labouring compared with white collar jobs, and higher injury severity compared with lower, were associated with reduced likelihood of RTW, which is as expected given physical function is an important requisite for many work tasks [21, 47, 52, 66]. Severe injuries were shown to influence return to full work duties more so than any RTW which may indicate a proportion of people with severe injuries returned to work in a modified capacity, which is a key component for the rehabilitation of injured workers [67].

Identification of modifiable risk factors is crucial for characterizing those at risk of poor RTW outcomes following injury and for targeted intervention programs [20]. Several psychometric assessment tools administered early post-RTI (less than one month) were associated with long-term work incapacity. Use of the NRS and PCS to assess baseline pain severity and pain-related catastrophizing were explanatory factors for delayed RTW in the short term only. Previous findings suggest that RTI survivors with post-traumatic stress had lower work capacity than those without post-traumatic stress [68]. Post-traumatic stress, evaluated using the IES-R in our study, was found to be highly correlated with DASS-21 scores which may account for the IES-R not appearing in the final explanatory model. The presence of a probable major depressive disorder (DASS-21 total score ≥ 15) was more sensitive for predicting delayed RTW than the previously mentioned tools, however, this association was restricted to any RTW. Vulnerable subgroups of people with persistent depressive mood symptoms are more likely to exhibit pain interference with daily functioning following RTI [4], which may partly explain this association being present longitudinally.

High pain-related disability and psychological distress, classified by an OMPSQ-SF score ≥ 50, were found to be important explanatory factors of RTW over time. Previous research has shown that this cut off was appropriate in predicting those at risk of poor recovery and delayed RTW up until 12-months post-injury, but not at 24-months [13, 69]. Gopinath et al. [13] noted that the attenuation of this association beyond 12-months may have been due to: i) inadequate statistical power; ii) the OMPSQ-SF being better suited to predicting poor recovery after back injury, and iii) psychological factors being of greater importance for predicting long term work incapacity. The sample size in our study may have been more appropriate to identify significant long-term associations between early high pain-related disability and psychological distress and RTW in a mixed injury cohort. Furthermore, the OMPSQ-SF has been shown to be effective in predicting poor recovery following whiplash injury [70], a common outcome of non-catastrophic road traffic crashes.

### Strengths and limitations

Several study limitations were considered when analysing and interpreting our findings. Multiple attempts were made to contact participants within the follow-up timeframes and several response modalities were implemented to minimize follow-up loss; phone interview, hard questionnaire, and online questionnaire. However, participants follow-up rates were shown to decline over time; 74% at 6-months, 59% at 12-months and 53% at 24-months. Identification of the reasons for loss to follow-up was not approved in the study protocol. Consequently, longitudinal models were used for the adjusted analyses to account for missing data due to loss to follow-up under a missing at random assumption. Data on treatment provided to participants and individual return to work policies were not collected, and would be valuable to examine further in future studies.

A key finding of the study was that claiming compensation within the prevailing fault-based scheme was associated with reduced likelihood of RTW. The Motor Accident Injuries Act 2017 (NSW) applies to claims arising from injuries sustained from 1 December 2017. This legislation reformed the NSW CTP compensation scheme, making it a substantially hybrid scheme with no-fault benefits for all claimants for six months after injury and fault-based benefits retained for claimants with injuries exceeding a minimum threshold of severity [71]. These reforms were driven in part by research evidence linking aspects of scheme design to the poorer outcomes experienced by claimants. While our findings about compensation-related effects on RTW are based on the experiences of a pre-reform cohort in NSW, they remain relevant to local and international injury compensation schemes engaged in the continual process of design, review and reform of law and practice in injury compensation schemes. Furthermore, several key modifiable factors associated with RTW are identified in this study (e.g., pain and psychological distress) after controlling for covariates, which included whether a person claimed compensation under the old scheme.

### Implications for future research, policy, and practice

Planned future work will: i) examine how impacts of the RTI on RTW immediately after the crash may influence claiming decisions, and how this differs from the effect of claiming itself on long-term work incapacity; ii) evaluate the effect of changes in the NSW compensation scheme on recovery trajectories and work outcomes, by comparing this cohort with a representative cohort of RTI participants under the new compensation scheme.

The findings of this paper support a focus on RTW as a post-crash response to prevent permanent disability; a key target of the World Health Organization Decade of Action for Road Safety 2021–2030 Global Plan [72]. Given that RTW rates post-RTI did not significantly change after 6-months, people recognized to have poor work capacity post-RTI should be the focus of RTW intervention programs. Workplace interventions carried out by healthcare professionals such as graded activity programs and work focussed cognitive behavioural therapy are effective in reducing time associated with work incapacity in musculoskeletal, pain-related, and mental health conditions [73]. Workplace policies could be revised to accommodate early and appropriate return of injured workers into modified duties at work. Employers who offer modified work programs can increase the likelihood of injured workers returning to work and reduce the number of absentee days [67]. Implementation of these programs, however, is dependent upon work capacity of the injured employee, the nature of the role, and financial factors. Employers could be encouraged to access financial support where possible to provide injured employees suitable RTW options. The current NSW CTP Recover at Work Assist program provides financial assistance to employers for up to 12-weeks to support employees, who have an accepted injury compensation claim, to recover at work [74].

While multi-domain programs involving healthcare provision and modified work duties are recommended [73], success of these programs hinge on appropriate policy settings and direct communication between healthcare providers, healthcare professionals, employers, and insurers [75]. Dedicated vocational rehabilitation personnel could be advocated for within RTW programs to educate employers on capabilities of the injured worker, facilitate communication between relevant parties involved, and provide workplace support to achieve successful RTW [76], however, their effectiveness and feasibility in supporting workers post-RTI requires further investigation.

Our findings about the association between with claiming compensation and RTW suggest that continued attention is required to the way scheme design, claims management and claims processes contribute to claimants’ experiences and outcomes. For instance, claimants in Victoria’s hybrid compensation scheme perceived that scheme as more fair, and exhibited greater recovery and health outcomes compared with those under the NSW fault-based scheme [63]. A high proportion of people with mild-to-moderate RTIs in a pilot NSW cohort reported unmet rehabilitation needs early after discharge from hospital [77]. Compensation scheme settings that support early access to appropriate rehabilitation providers should be advocated for, and the impacts on claimants’ health and social outcomes generated by features of claims processes including delays and disputes should be further explored [63].

At the level of clinical practice, our findings support early assessment of workers with RTIs using the OMPSQ-SF to stratify those at risk of poor prognosis and guide intervention programs. This process was shown to be effective for workplace injuries, where injured workers screened as high risk were offered psychological assessment and a multidisciplinary treatment program to address an individual’s barriers to returning to work [78]. Time away from work was more than halved with this approach compared to usual care and compensation claim costs were reduced by 30%. A similar implementation strategy could be applied in the context of RTIs. With reference to low- and middle-income countries, the findings of this study that post-crash psychological distress and higher levels of pain contribute to difficulty returning to work are applicable to those countries. Early clinical detection of people at risk of long-term work incapacity post-RTI is encouraged to strengthen professional medical care associated with the post-crash response, as recommended in the Decade of Action for Road Safety 2021–2030 Global Plan [72]. However, there are societal factors that are probably different in those countries and their effect on RTW rates are unknown.

## Conclusions

In our cohort of people with mild-to-moderate RTIs, one in five were in modified work duties or not working at all between six months to two years post-injury. We found that that injury compensation claimants within a fault-based road crash compensation scheme had poorer work outcomes compared with non-claimants, notably, with reduced likelihood for returning to full work duties over time. A range of early identified factors were found to be associated with long-term work incapacity, many of which are modifiable with intervention and can be assessed using easily administered clinical tools, such as, assessment of depressive mood using a validated screen like DASS-21, or pain-related disability using the OMPSQ-SF. Our findings add to previous Australian cohort studies in this research field, and we propose areas for practice change and targeted interventions to improve social and health outcomes following RTI, including review of compensation scheme design and claims management, multidisciplinary interventions for people at risk of long-term work incapacity, and the facilitation of early RTW.

## Data Availability

The datasets used and/or analysed during the current study are available from the corresponding author on reasonable request.

## Abbreviations

RTI: Road traffic injury
mTBI: Mild traumatic brain injury
RTW: Return to work
NSW: New South Wales
IRSAD: The Index of Relative Socio-economic Advantage and Disadvantage
EQ-5D-3L: Health related quality of life measure
BMI: Body mass index
ISS: Injury Severity Score
CTP: Compulsory third party
IES-R: Impact of Event Scale Revised
DASS-21: Depression and Anxiety Stress Scales
NRS: Numeric Rating Scale
OMPSQ-SF: Örebro Musculoskeletal Pain Screening Questionnaire Short Form
PCS: Pain Catastrophizing Scale
RR: Relative risk
CI: Confidence interval
